# Hydrogen-Induced Delayed Cracking in TRIP-Aided Lean-Alloyed Ferritic-Austenitic Stainless Steels

**DOI:** 10.3390/ma10060613

**Published:** 2017-06-03

**Authors:** Suvi Papula, Teemu Sarikka, Severi Anttila, Juho Talonen, Iikka Virkkunen, Hannu Hänninen

**Affiliations:** 1 Department of Mechanical Engineering, Aalto University School of Engineering, P.O. Box 14200, Aalto FI-00076, Finland; teemu.sarikka@aalto.fi (T.S.); iikka.virkkunen@aalto.fi (I.V.); hannu.e.hanninen@aalto.fi (H.H.); 2 Centre for Advanced Steels Research, University of Oulu, P.O. Box 4200, Oulu 90014, Finland; severi.anttila@oulu.fi; 3 Outokumpu Oyj, P.O. Box 245, FI-00181 Helsinki, Finland; juho.talonen@outokumpu.com

**Keywords:** ferritic-austenitic stainless steel, martensite transformation, hydrogen, delayed cracking, deep drawing, constant load tensile testing

## Abstract

Susceptibility of three lean-alloyed ferritic-austenitic stainless steels to hydrogen-induced delayed cracking was examined, concentrating on internal hydrogen contained in the materials after production operations. The aim was to study the role of strain-induced austenite to martensite transformation in the delayed cracking susceptibility. According to the conducted deep drawing tests and constant load tensile testing, the studied materials seem not to be particularly susceptible to delayed cracking. Delayed cracks were only occasionally initiated in two of the materials at high local stress levels. However, if a delayed crack initiated in a highly stressed location, strain-induced martensite transformation decreased the crack arrest tendency of the austenite phase in a duplex microstructure. According to electron microscopy examination and electron backscattering diffraction analysis, the fracture mode was predominantly cleavage, and cracks propagated along the body-centered cubic (BCC) phases ferrite and α’-martensite. The BCC crystal structure enables fast diffusion of hydrogen to the crack tip area. No delayed cracking was observed in the stainless steel that had high austenite stability. Thus, it can be concluded that the presence of α’-martensite increases the hydrogen-induced cracking susceptibility.

## 1. Introduction

Dual-phase materials can combine the beneficial properties of the constituent phases. Ferritic-austenitic (duplex) stainless steels possess an excellent combination of mechanical properties, e.g., high yield strength, and good corrosion resistance. The presence of two phases and their synergism induces strengthening due to grain refinement and high volume fraction of interphase boundary. There is potential for increased ductility in duplex stainless steels if the austenite phase is metastable. Deformation-induced martensite transformation enhances the work-hardening rate of the material, resulting in favorable combination of strength and elongation. Lean metastable duplex stainless steels, with lower alloying additions and lower material cost in comparison to conventional duplex steel grades, are attractive materials for many applications, e.g., in the construction industry.

In metallic materials, high strength generally means increased susceptibility to hydrogen embrittlement (HE). Hydrogen embrittlement is a process resulting in a decrease of toughness, ductility, and load-bearing capacity of a material. Hydrogen degradation, representing one of the main limitations to demanding applications of advanced high-strength steels, can be classified into several forms, e.g., internal hydrogen embrittlement due to absorbed hydrogen in the material and hydrogen environment embrittlement due to exposure of a material to hydrogen during service [1,2]. HE phenomena typically depend on three essential factors: the presence of hydrogen, tensile stress (applied or residual), and an inappropriate microstructure [3]. The effects of hydrogen on metals can range from a slight decrease in ductility to brittle macroscopic fracture at relatively low applied stress, often below the yield strength. HE mechanisms are governed by local hydrogen redistribution within the material. Highly stressed areas are subject to a lattice distortion increasing local hydrogen solubility and thus a chemical potential gradient acting as the driving force for hydrogen diffusion [4]. Thus, areas with high residual stresses are subjected to local hydrogen accumulation, dramatically increasing the susceptibility to delayed cracking, a subcritical crack growth mechanism. Hydrogen diffusivity and solubility are key parameters in delayed cracking. 

Hydrogen may enter steels during the production operations or during service environment exposure by the absorption of hydrogen atoms from dissociation of hydrogen-containing gases or by hydrogen atoms produced in electrochemical reactions in a solution. Hydrogen can be absorbed during melting and casting operations from water contained in the raw materials or in the furnace gases; from acidic pickling and electrolytic cleaning solutions; or during bright annealing, electroplating, or welding. Additionally, hydrogen can be generated by in-service corrosion, galvanic interaction between dissimilar metals or cathodic protection.

Duplex stainless steels are generally more susceptible to hydrogen embrittlement than austenitic stainless steels, due to the presence of the ferrite phase. Hydrogen diffusivity and permeation are markedly higher in ferritic structure (BCC crystal structure) in comparison to austenite face-centered cubic (FCC). Thus, the transport of hydrogen through a duplex steel occurs mainly through the ferrite phase [5]. Hydrogen solubility in ferrite is, however, much lower than that in austenite. The amount of hydrogen trapped in the ferritic and austenitic microstructures, and also in the large interfacial area between the phases, is a significant factor in hydrogen embrittlement susceptibility. Hydrogen weakens the strength of various interfaces in metals and alloys, and hydrogen-assisted cracking is often observed along grain boundaries, phase boundaries etc. Ferrite suffers from more extensive embrittlement than the austenite phase and provides easier crack initiation, but the islands of austenite can act as effective barriers to crack propagation [6]. The shape, size, and spacing of the austenite islands influence the hydrogen trapping tendency and crack arrest properties of the steel [7]. 

Hydrogen effects on the mechanical properties of each phase in a multiphase material are strongly coupled with existing residual stresses in the microstructure [8]. Thermal stresses are formed in duplex stainless steels during cooling from the solution-annealing temperature, since the two phases have different coefficients of thermal expansion. Residual tensile stresses arise in the austenite phase with a higher coefficient and balancing compressive stresses arise in the ferrite phase [9]. In addition, residual stresses are induced in a material during various forming operations. Residual stresses are a consequence of interactions between deformation, temperature, and microstructure [10]. The difference in the actual strain level in different locations may be caused by several reasons, including a difference in strength between the co-existent phases in the material, due to die/mold shape or constraints from the gripping force on the workpiece, or by temperature gradients [10]. 

The stability of the austenite phase is another important factor in HE resistance of duplex stainless steels [6]. There is growing interest in metastable duplex steels, utilizing the TRIP effect for increased strength and elongation. It has been found that even 0.30 volume fraction of metastable austenite seems efficient for achieving improved ductility in ferritic-austenitic stainless steels [11]. However, transformation of austenite to α’-martensite causes volumetric expansion and local stress concentrations. It has been shown that the γ→α’ transformation markedly increases the magnitude of total residual stresses in deep-drawn metastable austenitic stainless steels [12]. Hydrogen diffusivity is high in α’-martensite, having a BCC crystal structure, offering a fast pathway for hydrogen to potential crack initiation sites, and susceptibility of the steel to hydrogen embrittlement increases because of the presence of α’-martensite phase [13,14]. However, it is hard to find any published studies on the effect of strain-induced martensite transformation on hydrogen embrittlement and delayed cracking of lean duplex stainless steels.

In this study, the potential susceptibility of three lean-alloyed metastable TRIP-aided ferritic-austenitic stainless steels to hydrogen-induced delayed cracking after plastic forming was investigated. The research concentrated on the effects of internal hydrogen present in the materials after production operations.

## 2. Results

**Figure 1:**
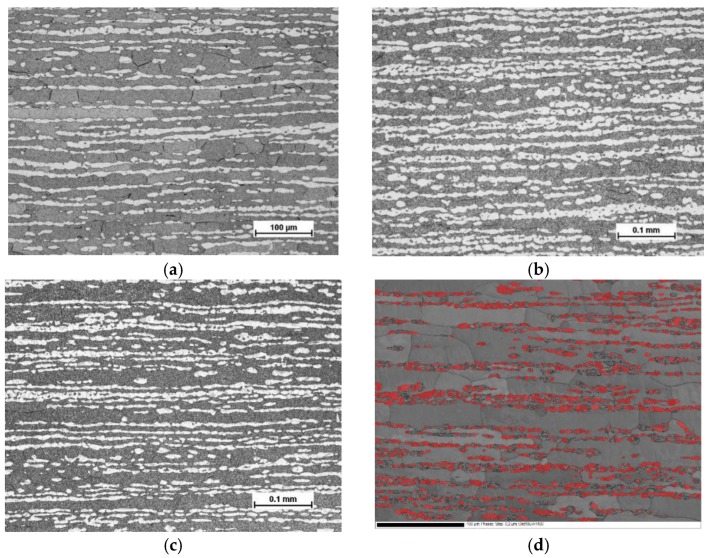
Microstructure of the studied materials: (**a**) steel A; (**b**) steel B; (**c**) and (**d**) steel C.

Optical micrographs of the test materials, taken from a cross-section of rolling vs. normal direction of the cold-rolled sheets, are presented in Figure 1a–c. The Beraha etchant colored the ferrite phase darker and left the austenite phase lighter. In Figure 1d, a SEM backscattering electron image revealing grain and phase boundaries, and austenite phase colored with red, is presented for the steel C. It is evident that despite careful specimen preparation and electro-polishing, mechanical grinding has induced martensite transformation in some austenitic areas. According to the EBSD characterization, grain size of ferrite was markedly larger than that of austenite, particularly along the rolling direction.

**Figure 2:**
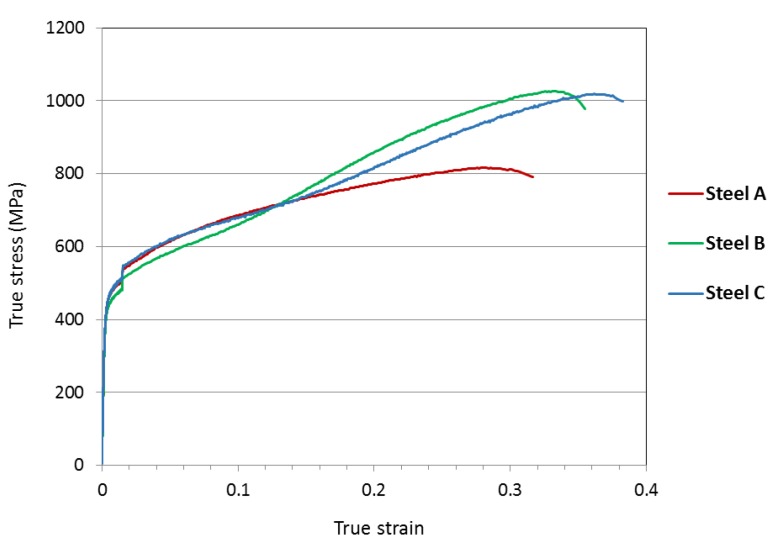
Tensile true stress–true strain graphs for the studied materials.

The measured true stress–true strain curves for the test materials, tested in uniaxial tensile loading along the rolling direction, are presented in Figure 2. The presented data is an average of three specimens for each material. The step in the curves soon after the yield point is due to the change of strain rate after 1.5% strain. A distinct increase in the slope is seen in the curves of steel B and steel C after about 0.13 true strain, which indicates increased strain hardening rate. Tensile properties of the studied materials, and standard deviation, are presented in Table 1. Markedly higher elongation and ultimate tensile strength were attained in steels B and C in comparison to steel A.

The content of ferromagnetic phases in the test materials, according to Satmagan measurements, is presented in Table 2, both before and after the tensile testing. The volume fraction of ferrite phase remained constant, so the difference between the Satmagan reading at the initial stage and after tensile straining represents the fraction of α’-martensite. The ferrite content in all the test materials was about 70% in the initial stage. During tensile testing, a considerable fraction of the austenite phase was transformed into martensite in steels B and C, whereas in steel A austenite had high stability. 

**Figure 3:**
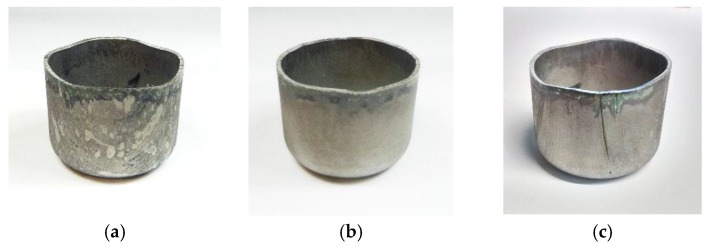
Deep-drawn cups at DR 2.0: (**a**) steel A; (**b**) steel B; and (**c**) steel C.

Swift cup testing for these materials was successful up to drawing ratio of 2.0. At drawing ratios 2.1 and above, the cups cracked in the most thinned area near the punch radius. In Swift deep drawing testing, there was no delayed cracking occurring in the cups of steel A and steel B at drawing ratios 1.8 and 2.0. However, in steel C an individual crack was observed in one of the cups at drawing ratio 2.0 within 0.5 h from the forming operation. Pictures of Swift cups at DR 2.0 are presented in Figure 3.

**Figure 4:**
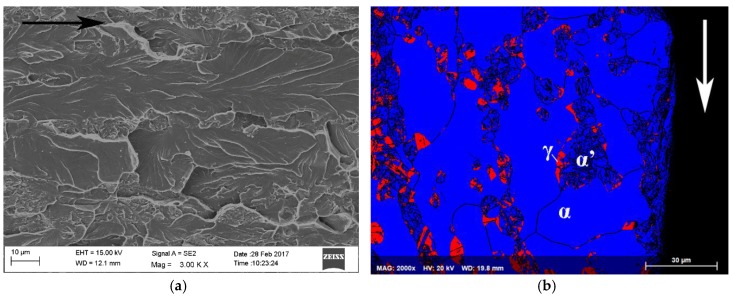
Fracture surface (**a**) and EBSD phase map (**b**) of the delayed crack in the cup of steel C at DR 2.0. The direction of crack propagation is marked with arrows.

Fracture surface of the delayed cracking in steel C cup is presented in Figure 4a. Crack initiation occurred at the upper edge of the cup. The fracture mode was transgranular cleavage. Large faceted cleavage fracture dominated in the ferrite phase, and quasi-cleavage fracture associated with slight plastic deformation was observed in the austenite phase. An EBSD phase map from the cross-section of the fracture surface is presented in Figure 4b. Austenite (FCC) phase is colored with red and ferrite (BCC) and α’-martensite (BCC) phases with blue. Austenitic areas have been partly transformed to α’-martensite, especially in the immediate vicinity of the crack edge. According to Satmagan measurements, average austenite content at this location in the cup wall was 8%.

Constant load tensile testing was conducted on specimens with pre-straining: 0.27 engineering strain for steel A and 0.31 for steels B and C. The results of the tests are presented in Table 3. In the constant load tensile testing, delayed cracking was observed in some specimens of steels B and C at high applied stress ratios, above 0.92*NTS. No cracking occurred in steel A. 

**Figure 5:**
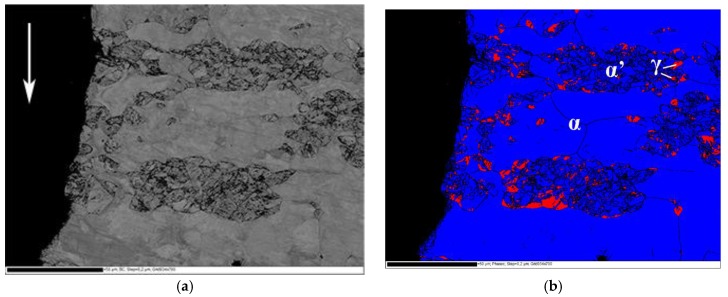
A backscattering electron image (**a**) and EBSD phase map (**b**) of the fracture path in steel B constant load specimen. The direction of crack propagation is marked with an arrow in (**a**).

A backscattering electron image and EBSD phase map from the cross-section of the fracture surface of the constant load tensile test specimen of steel B tested at applied stress ratio 0.945 are presented in Figure 5a,b. Austenite (FCC) phase is colored with red and BCC-phases ferrite and α’-martensite phase with blue. The microstructure in the immediate vicinity of the fracture surface consisted almost completely of BCC structure, so cracking seemed to propagate along the ferrite and α’-martensite phases.

**Figure 6:**
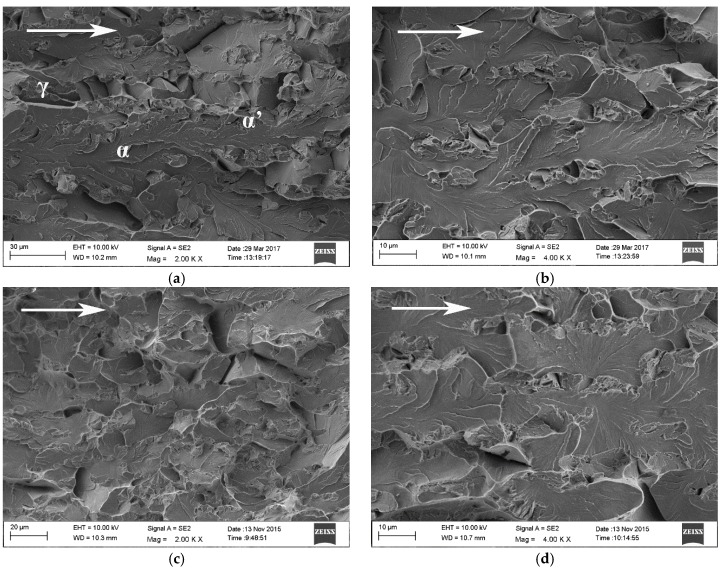
Fracture surfaces of constant load tensile specimens of steel B (**a**,**b**) and steel C (**c**,**d**).

Fracture surface of steel B constant load tensile specimen that broke after 103 h is presented in Figure 6a,b and that of steel C constant load tensile specimen that broke after 241 h in Figure 6c,d. The direction of crack propagation is marked in the figures with arrows. The fracture mechanism in both materials was predominantly cleavage along the ferrite phase. The regions of prior austenite, transformed mostly to α’-martensite during the pre-straining of the specimens and crack propagation, are clearly distinguishable on the fracture surfaces. Delayed fracture in the constant load tensile test specimens initiates at the notch root. A flat triangular area, associated with high stress triaxiality, is typically visible on the fracture surface. 

## 3. Discussion

According to the deep drawing and constant load tensile testing, the studied materials seem not to be particularly susceptible to delayed cracking. For example, in the Swift cup tests cracking was only observed in one cup of steel C at the highest drawing ratio 2.0. The crack was suspected to have initiated from a stress concentrator, possibly a local material inhomogeneity or poor edge quality of the Swift cup blank. The Swift cup test is commonly used in steel industry for evaluating formability of different steels and their possible susceptibility to delayed cracking. It provides a convenient way of comparing the performance of various sheet metals. However, the exact same stress state is not necessarily reproduced in repeated tests. 

By constant load tensile testing, it is possible to conduct more systematical examination of the effect of different factors on delayed cracking. In constant load tensile testing, delayed cracking was observed in some specimens at high applied stress ratios, above 0.92*NTS. The most frequent cracking was observed in steel B. This was the material where the austenite was most unstable, i.e., the highest volume fraction of strain-induced α’-martensite was present in the pre-strained specimens. No delayed cracking occurred in steel A, in which the austenite phase was highly stable. So, it seems that the presence of α’-martensite was a necessary prerequisite for delayed cracking to occur, similarly to austenitic stainless steels [15].

Initiation of delayed cracking in the studied materials requires the presence of a stress concentrator, which will accumulate a local high concentration of hydrogen. In steel C, there was only one occasion of cracking both in Swift cup tests and in constant load tensile testing. Hydrogen-induced cracking initiation is dependent on the presence of localized trapping sites, such as non-metallic inclusions in high-strength steels, and the probability of their location at critical areas, such as the notch root. The studied materials were pilot-mill processed, and the surface quality was not as uniform as in industrial production materials. The hydrogen content in the steels varied between 1.9–3.0 wppm, which is somewhat lower than a typical level in austenitic stainless steels, due to higher hydrogen solubility in austenite than in ferrite. However, ferrite can be embrittled with very low levels of hydrogen present [6].

The constant load tensile tests were performed using standardized (ASTM E292-09) notched specimens. The notches were prepared by electric discharge machining, in which the high spark temperature causes localized melting in a thin surface layer (1–3 µm). It is possible that some hydrogen absorption into the metal from the deionized water occurs during the process. However, it was not possible in this study to determine the local hydrogen content at the notched region.

Cold-rolled duplex stainless steels exhibit a strong anisotropy due to their two-phase microstructure. During the industrial rolling process, both phases become elongated in the rolling direction, and also clear and intense crystallographic rolling textures develop, especially in the ferrite phase [16,17]. As a result of this anisotropy, the mechanical properties of rolled duplex stainless steels are strongly dependent on direction. High planar anisotropy induces inhomogeneous deformation during cup forming, leading to a localized plastic strain and the concentration of residual stresses in certain areas such as rim areas of the cup [18].

It is well known that increasing the volume fraction of strain-induced α’-martensite lowers the resistance of metastable austenitic stainless steels to hydrogen embrittlement [13,19]. Hard martensite microstructure is more sensitive to the presence of hydrogen than that of austenite. Recently, it was shown that the martensite transformation route has a marked effect on hydrogen embrittlement of TRIP-assisted duplex stainless steels; direct γ→α’ transformation results in higher strain incompatibility between the phases, whereas multi-stage γ→ε→α’ transformation with more compatible strain evolution and weaker localization of plastic deformation in ferrite increases the resistance against hydrogen-induced crack initiation and growth [20]. In the studied stainless steels, almost negligible volume fraction of ε-martensite was detected in EBSD-analysis of specimens strained to 0.15 engineering strain, and therefore the transformation route is likely to be the direct γ→α’ transformation. 

In stainless steels with metastable austenite, once a delayed crack has initiated, crack propagation is facilitated by strain-induced α’-martensite transformation in the highly plastically deformed region ahead of the crack tip. Localized martensite transformation and crack propagation along α’-martensite phase have been reported in several hydrogen embrittlement studies of austenitic stainless steels [21,22,23]. The local martensitic transformation and high dislocation density enhances hydrogen entry and trapping in the region [24]. 

The distribution of the phases in duplex stainless steels plays a significant role in crack propagation through hydrogen embrittled material [25]. In a strongly banded duplex microstructure the orientation of the austenite islands perpendicular to the crack propagation direction promotes effective crack arrest. In this study, the constant load tensile testing was conducted in the rolling direction of the material, i.e., the delayed crack initiating from the notch root propagated perpendicular to the banded dual-phase microstructure. However, when the austenite was metastable, it was transformed to strain-induced α’-martensite, which facilitated hydrogen diffusion and decreased the crack arrest tendency of the austenite phase in the duplex microstructure and the cracks propagated easily through ferrite and martensite. 

## 4. Materials and Methods 

The studied materials were three lean-alloyed pilot-scale ferritic-austenitic stainless steels. The steels had very low levels of nickel, which was partly replaced by manganese and nitrogen. They were studied in cold-rolled and solution-annealed (1050 °C/5 min) condition. The thickness of the sheets was 1.5 mm. The chemical composition of the test materials is presented in Table 4. Hydrogen content was analyszed with melt extraction using a Leco TCH 600 NOH equipment (LECO Corp., Saint Joseph, MI, US).

The microstructure of the test materials was studied by optical microscopy with Nikon Epiphot 200 microscope (Nikon Instruments, Tokyo, Japan). The specimens were ground up to 2500 grit with SiC abrasive papers and then electro-polished with A2 electrolyte at 35 V for 20–25 s. A modified Beraha etchant (1.0 g K_2_S_2_O_5_, 15 mL HCl, and 85 mL H_2_O) was used to reveal the two-phase microstructure.

The austenite phase in the studied stainless steels is designed to be metastable in order to utilize the TRIP effect for improved combination of strength and elongation. The content of the ferrite phase in the initial state and the transformed α’-martensite in the deformed specimens was measured with a Satmagan equipment (Rapiscan Systems, Torrance, CA, US). Satmagan is a magnetic balance measurement device that is used to determine the content of the ferromagnetic phase in a specimen (size 6 × 15 mm^2^). In a Satmagan measurement, a saturating magnetic field is applied to the specimen that is placed in a sample holder. The magnetic field causes a force that is recorded by adjusting a potentiometer. The relation between the potentiometer reading *S* and the total content of the ferromagnetic phases is expressed as [26]

(1) $$S=K^{*}\left(C_{\mathrm{fm}}{}^{*}M_{\mathrm{sat}}\right)/\rho ,$$

where *K* is a constant, *C*_fm_ is the content of the ferromagnetic phases, *M*_sat_ is the saturation magnetization of the ferromagnetic phase and *ρ* is density. The value of constant *K* is determined by empirical calibration. In this investigation the calibration constant determined by Rintamaa [26] was used.

Uniaxial tensile testing was performed with a MTS 810 servo-hydraulic material testing system (MTS, Eden Prairie, MN, USA) and a MTS 632.12C-20 extensometer (MTS, Eden Prairie, MN, USA) at ambient temperature. According to standard SFS-EN ISO 6892-1, the initial stain rate was 1.1 mm/min until 1.5% strain, after which the strain rate was increased to 30.2 mm/min. Three specimens were tested for each material.

Swift cup forming tests, measuring the formability of materials under press-forming operations, were carried out with an Erichsen testing machine (ERICHSEN GmbH & Co. KG, Hemer, Germany). Laser cut circular steel blanks were deep drawn into cups by a flat-bottomed cylindrical punch with 50 mm diameter. Novacel lubricant and blank holder force of 25 kN were used. Swift cup tests are used for determining the limiting drawing ratio, i.e., the maximum blank diameter that can be successfully drawn divided by the punch diameter. The tests were made at room temperature. The appearance of cracks was visually examined after 0.25, 0.5, 1, 2, 4, 8, 24, 48, 72, and 120 h from the drawing operation.

Constant load tensile testing was conducted using a prior developed testing arrangement [27]. Specimens were first pre-strained to certain strain levels (0.27–0.31) to simulate the conditions after forming operations and to produce strain-induced α’-martensite in the material. Pre-straining and constant load testing was done with a MTS Insight 30 kN electromechanical tensile tester. In order to produce a stress concentrator and multi-axial stress state in the constant load tensile test specimens, notched specimens were used, according to standard ASTM E292-09. The notched tensile specimens had a 60° double-edge notch with a root radius 0.3 mm, producing a stress concentration factor *k*_t_ = 4.5. The notches were prepared using electric discharge machining. Each test was performed so that the notches were machined directly after pre-straining of the specimen, and the constant load experiment was started within one hour from pre-straining. The applied stress ratio for each constant load test was defined as the applied stress divided by the ultimate tensile strength, or the notched tensile strength (NTS), of a notched tensile test specimen.

Fracture surfaces of the Swift cups and constant load tensile specimens were examined with FEG-SEM Zeiss Ultra 55 field emission gun scanning electron microscope (FEG-SEM) (Carl Zeiss Microscopy GmbH, Münich, Germany). Electron backscattering diffraction (EBSD) measurements for identifying the FCC and BCC phases in the microstructure were done with an Oxford Nordlys F+ EBSD system. The EBSD data acquisition and analysis were performed using the HKL Channel 5 software from Oxford Instruments (version 5.0.9.0, Oxford Instruments, Abingdon, UK) .

## 5. Conclusions

The potential susceptibility of three lean-alloyed ferritic-austenitic stainless steels to hydrogen-induced delayed cracking was examined. Delayed cracking only occurred in two of the steels, the TRIP-aided ones with metastable austenite, at high stress levels, and initiated in the presence of a stress concentrator. No hydrogen-induced delayed cracking was observed in steel A, in which no martensite transformation occurred during straining/forming. Thus, it was demonstrated that the presence of martensite increases the hydrogen-induced cracking susceptibility. In the steels with metastable austenite, in highly plastically deformed areas austenite was transformed to α’-martensite, decreasing the crack arrest tendency of the austenite in the duplex microstructure. The fracture mode was predominantly cleavage along the BCC phases ferrite and α’-martensite and somewhat more ductile quasi-clevage in the remaining austenite phase.

## Figures and Tables

**Table 1:** Tensile properties of the studied materials.

Material	Yield Strength (MPa)	Ultimate Tensile Strength (MPa)	Uniform Elongation (%)	Elongation at Fracture (%)
Steel A	448 ± 1.2	634 ± 3.3	23.0 ± 1.3	34.0 ± 1.6
Steel B	430 ± 3.5	746 ± 4.1	36.0 ± 1.4	38.8 ± 1.3
Steel C	444 ± 4.9	718 ± 13.1	38.8 ± 1.7	40.0 ± 2.3

**Table 2:** Volume fraction of ferromagnetic phases in the test materials.

Material	Steel A	Steel B	Steel C
Intial stage	0.72	0.69	0.68
After tensile testing	0.73	0.94	0.88

**Table 3:** Time to fracture under constant tensile load.

Applied Stress Ratio	Time to Fracture Under Constant Load (h)		
	Steel A	Steel B	Steel C
0.97	600*→*	0.1	-
0.96	700→	2.1	600*→*
0.945	-	103	700*→*
0.93	-	600→	241
0.92	-	-	750→

**Table 4:** Chemical composition of the studied materials.

Material	C	Cr	Ni	Mn	Si	Mo	N	H
Steel A	0.018	21.9	0.1	3.0	0.32	0.21	0.181	3.0 wppm
Steel B	0.021	20.4	0.1	1.9	0.29	0.19	0.167	2.9 wppm
Steel C	0.015	20.4	0.2	2.9	0.30	0.19	0.159	1.9 wppm
